# Initiation of High‐Potency Benzodiazepine Prescriptions Among Survivors of Severe Trauma

**DOI:** 10.1111/aas.70245

**Published:** 2026-04-30

**Authors:** Anders Oldner, Mikael Eriksson, Emma Larsson, Jesper Eriksson, Erik von Oelreich

**Affiliations:** ^1^ Perioperative Medicine and Intensive Carse Karolinska University Hospital Stockholm Sweden; ^2^ Section of Anesthesiology and Intensive Care Medicine, Department of Physiology and Pharmacology Karolinska Institutet Stockholm Sweden; ^3^ Department of Anaesthesia, Operation and Intensive Care Uppsala University Hospital Uppsala Sweden; ^4^ Department of Surgical Sciences, Anaesthesia and Intensive Care Uppsala University Uppsala Sweden

## Abstract

**Background:**

Trauma is a major public health concern that often leads to long‐term psychological distress and chronic pain. Benzodiazepines (BZDs) are sometimes prescribed for anxiety, insomnia, or acute stress‐related symptoms, but long‐term use is associated with dependence and adverse outcomes. The extent to which BZDs are initiated after trauma, and their implications for long‐term health, remain poorly understood. This study aimed to assess the association between trauma exposure and initiation of high‐potency BZDs, identify risk factors within the trauma cohort and examine the association between new BZD use and long‐term mortality.

**Methods:**

We conducted a population‐based cohort study using data from a regional trauma registry linked to Swedish national health registers. New initiation of BZD prescriptions was defined as filling at least one prescription within 6 months after trauma. Multivariable logistic regression was used to assess associations between trauma exposure and BZD initiation and to identify risk factors within the trauma cohort. Cox proportional hazards regression evaluated the association between new BZD use and 6–18‐month mortality.

**Results:**

The study included 12,206 BZD‐naive trauma patients and 66,801 matched controls. Trauma exposure was independently associated with new high‐potency BZD use. Within the trauma cohort, risk factors included older age, psychiatric comorbidity, substance abuse, pre‐traumatic opioid or sedative‐hypnotic drug use, penetrating trauma, and higher injury severity. New BZD use was associated with markedly elevated 6–18‐month mortality (adjusted HR 2.9, 95% CI 2.0–4.2, *p* < 0.001), a finding that reflects the complex clinical and psychosocial vulnerability of this group.

**Conclusions:**

Trauma exposure independently predicted initiation of high‐potency BZDs among previously BZD‐naive patients. Psychiatric comorbidity, substance use, and greater injury severity were important risk factors. The association between new BZD use and increased long‐term mortality underscores the need for cautious prescribing and structured follow‐up after trauma.

**Editorial Comment:**

This study examines initiation of high‐potency benzodiazepines among previously naive survivors of severe trauma using linked registry data. It shows that trauma exposure is strongly associated with new benzodiazepine use, particularly in older, comorbid, and vulnerable patients. Initiation is also associated with higher subsequent mortality, likely reflecting underlying clinical and psychosocial risk rather than a causal drug effect.

## 1 Introduction

Traumatic injuries constitute a major global health challenge, contributing substantially to morbidity, long‐term disability, and premature mortality [1, 2]. Survivors of major trauma often experience a wide range of physical and psychological sequelae, including chronic pain, post‐traumatic stress disorder (PTSD), anxiety, and depression [3, 4, 5]. The management of these post‐trauma symptoms frequently involves pharmacological treatment.

Benzodiazepines (BZDs) are central nervous system depressants commonly prescribed for anxiety, insomnia, and muscle tension [6, 7, 8] Although they can be effective for short‐term symptom relief, clinical guidelines generally recommend caution regarding long‐term use due to risks of dependence, tolerance, cognitive impairment, and accidents or overdose [9, 10, 11]. Consequently, chronic BZD treatment is typically restricted to limited indications, and non‐pharmacological interventions or alternative medications are often preferred [12]. Thus, initiation of BZD treatment, particularly with high‐potency agents, may serve as a clinically relevant marker carrying the potential for adverse outcomes.

The trauma population may be particularly susceptible to BZD‐related risks, as these patients often exhibit higher rates of psychiatric comorbidity and substance use disorders [13, 14]. Previous research has demonstrated an association between trauma exposure and subsequent substance use; however, detailed information on the initiation of high‐potency BZD use among individuals confirmed as BZD‐naive before trauma remains limited [15]. Furthermore, prior evidence suggests that new‐onset BZD use may be linked to adverse long‐term outcomes, including increased mortality [16].

This study aims to address these knowledge gaps using a matched cohort design that combines data from a regional trauma registry with national Swedish health registers. Using a matched cohort design linking a regional trauma registry to national health registers, we aimed to assess the association between trauma exposure and initiation of high‐potency BZD prescriptions among previously BZD‐naive individuals, to identify clinical and demographic risk factors for new BZD use within the trauma cohort, and to characterize long‐term mortality in this patient group.

By elucidating patterns of BZD initiation following trauma and identifying patient groups at elevated risk, the findings may contribute to a better understanding of post‐traumatic pharmacological management and facilitate more individualized, cautious prescribing practices.

## 2 Methods

Ethical approval was obtained from the Regional Ethical Review Board in Stockholm, Sweden (approval number 2015/1137–31/4, date 2015‐08‐12 and amendment approval number 2018/751–32, date 2018‐04‐09), which waived the requirement for individual informed consent. The study has been performed in accordance with the ethical standards laid down in the 1964 Declaration of Helsinki and its later amendments. All analyses were conducted in compliance with national research and data protection regulations. The study was reported in accordance with the STROBE (Strengthening the Reporting of Observational Studies in Epidemiology) guidelines for observational studies [17].

### 2.1 Study Design and Setting

This population‐based matched cohort study was conducted using data from a regional trauma registry linked to multiple Swedish national health registers. The cohort comprised all trauma patients admitted to the Karolinska Trauma Center, the regional referral center for severe trauma in the Stockholm region, between 2006 and 2015. The center manages approximately 2 million inhabitants and serves as the tertiary referral unit for all major trauma cases in the region.

### 2.2 Study Population

Trauma patients aged ≥ 15 years admitted through the trauma unit, regardless of Injury Severity Score (ISS), were eligible. Patients admitted without trauma team activation but later found to have an ISS above threshold were also included in the registry. Individuals with isolated limb fractures, drowning, chronic subdural hematoma, burns, or hypothermia without trauma were excluded. Controls were drawn from the Total Population Register in a 1:5 ratio, matched by age, sex, and municipality of residence at the date of the index trauma. Patients dying within 3 months after trauma were excluded since they largely represent patients with fatal injuries who would not survive long enough to initiate outpatient prescribing.

### 2.3 Data Sources and Linkage

Data linkage was performed using the unique Swedish personal identification number, which allows individual‐level tracking across national health and population registers [18].

Information on BZD prescriptions was obtained from the Swedish Prescribed Drug Register [19] Benzodiazepines (BZDs) and related GABA‐A agonists (ATC codes N05BA, N05BC, N05CD, N03AE01) were classified as the primary drug group of interest. A comparator group of sedative‐hypnotic drugs, including antihistamines, melatonin receptor agonists, and other sedatives (ATC codes N05BB, N05BE, N05CC, N05CF, N05CH, N05CM, N05CX, R06A), was used as a covariate (see Table S1).

Comorbidity data up to 8 years before the trauma were retrieved from the National Inpatient and Outpatient Registers and categorized according to international classification of Diseases (ICD)‐10 codes. The Charlson Comorbidity Index (CCI) was used to quantify overall somatic comorbidity. Psychiatric comorbidity and substance use disorders were identified using established ICD‐10 groupings.

Educational level was obtained from the Longitudinal Integration Database for Health Insurance and Labour Market Studies (LISA) [20] and categorized as low (≤ 9 years), medium (10–12 years), or high (> 12 years). Injury severity was quantified using the ISS, while severe injury to a specific body region was defined as an Abbreviated Injury Scale (AIS) score > 2. Shock on arrival was defined as a systolic arterial pressure (SAP) < 90 mmHg, and the Glasgow Coma Scale (GCS) score was also recorded. Mortality data for the period 6–18 months post‐trauma (the secondary outcome) were obtained from the Swedish Cause of Death Register [21].

### 2.4 Definitions of Benzodiazepine Use and Outcome

Pre‐traumatic use of benzodiazepines or sedative‐hypnotic drugs was defined as filling at least one prescription for the respective drug type within the 6 months preceding the trauma event. Patients were considered BZD‐naive if they had not filled any prescription for high‐potency benzodiazepines during that period, regardless of low‐potency use.

The primary outcome was defined as new initiation of BZD prescriptions, defined as filling at least one prescription within 6 months after trauma.

### 2.5 Statistical Analyses

Categorical variables were presented as counts and percentages, and continuous variables as medians with minimum and maximum values, or means with standard deviations, as appropriate. Between‐group comparisons were performed using the Chi‐squared test for categorical variables and the Mann–Whitney U‐test for continuous variables.

Multivariable logistic regression was applied to estimate odds ratios (ORs) for the association between trauma exposure and new post‐traumatic high‐potency BZD use, adjusting for potential confounders. The full model included age, sex, income, education, somatic comorbidity (CCI), psychiatric comorbidity, substance abuse, pre‐traumatic opioid use, and pre‐traumatic sedative‐hypnotic drug use. Because matching was not always complete, particularly for elderly patients in smaller municipalities, age and sex were retained as covariates in all regression models to adjust for any residual imbalance. Additional multivariable analyses were conducted within the trauma cohort to identify independent risk factors for new BZD use.

Cox proportional hazards regression models were used to estimate hazard ratios (HRs) for the association between new BZD use and mortality during the 6–18‐month follow‐up, adjusting for age, sex, comorbidities, and injury severity (ISS). A sensitivity analysis additionally adjusting for severe AIS‐region injuries (head, thorax, and spine, AIS > 2) was conducted to evaluate residual injury‐severity confounding. A sensitivity analysis excluding deaths within 6 months (rather than 3 months) assessed the impact of the primary exclusion criterion. A sensitivity analysis employing probability weights was performed to account for potential bias related to non‐random dropout due to death [22].

All statistical tests were two‐sided, and a *p* value < 0.05 was considered statistically significant. All analyses were performed using Stata Statistical Software (StataCorp LLC, College Station, TX, USA).

## 3 Results

**FIGURE 1:**
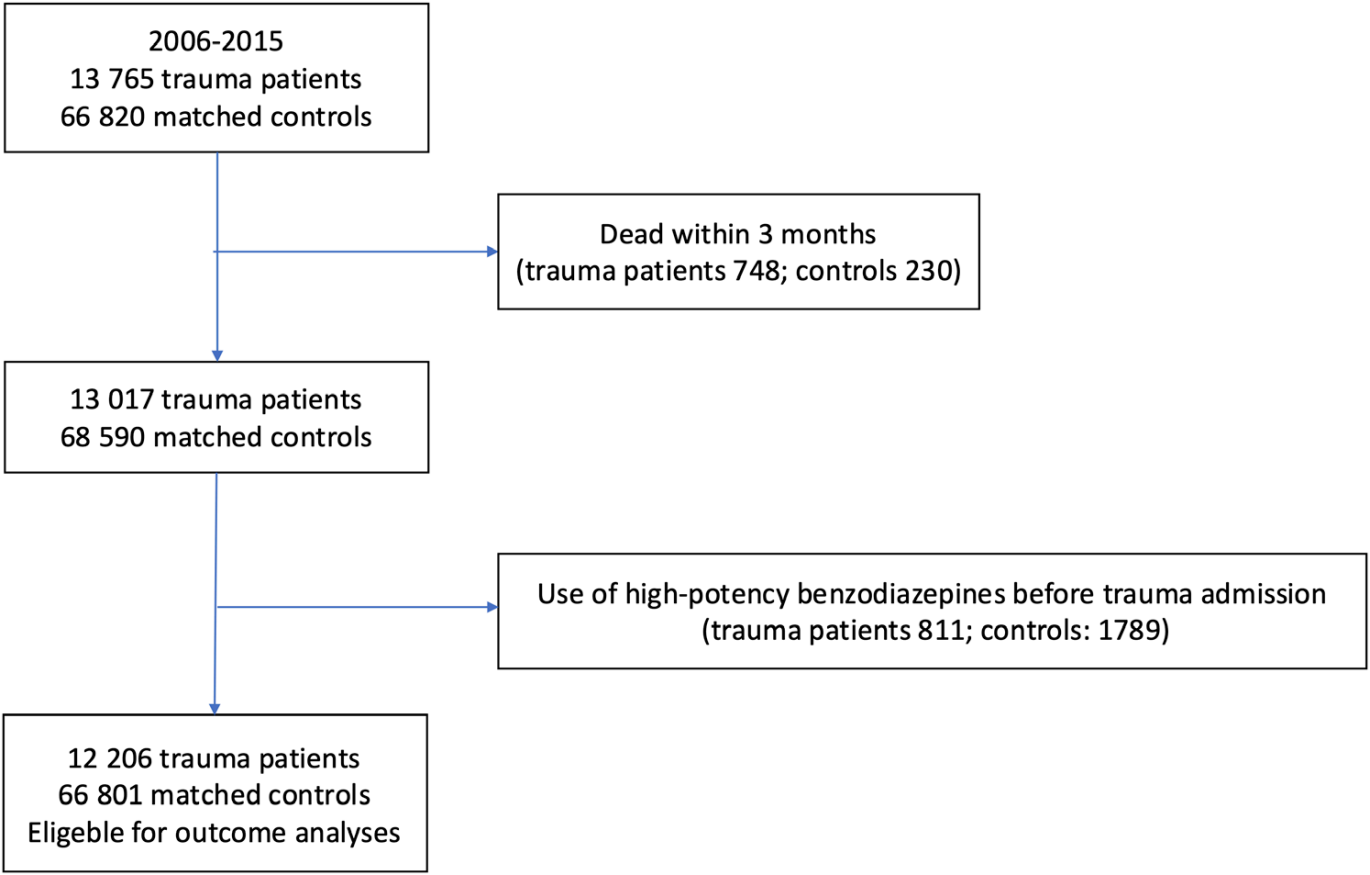
Flow chart of included patients and matched controls.

**FIGURE 2:**
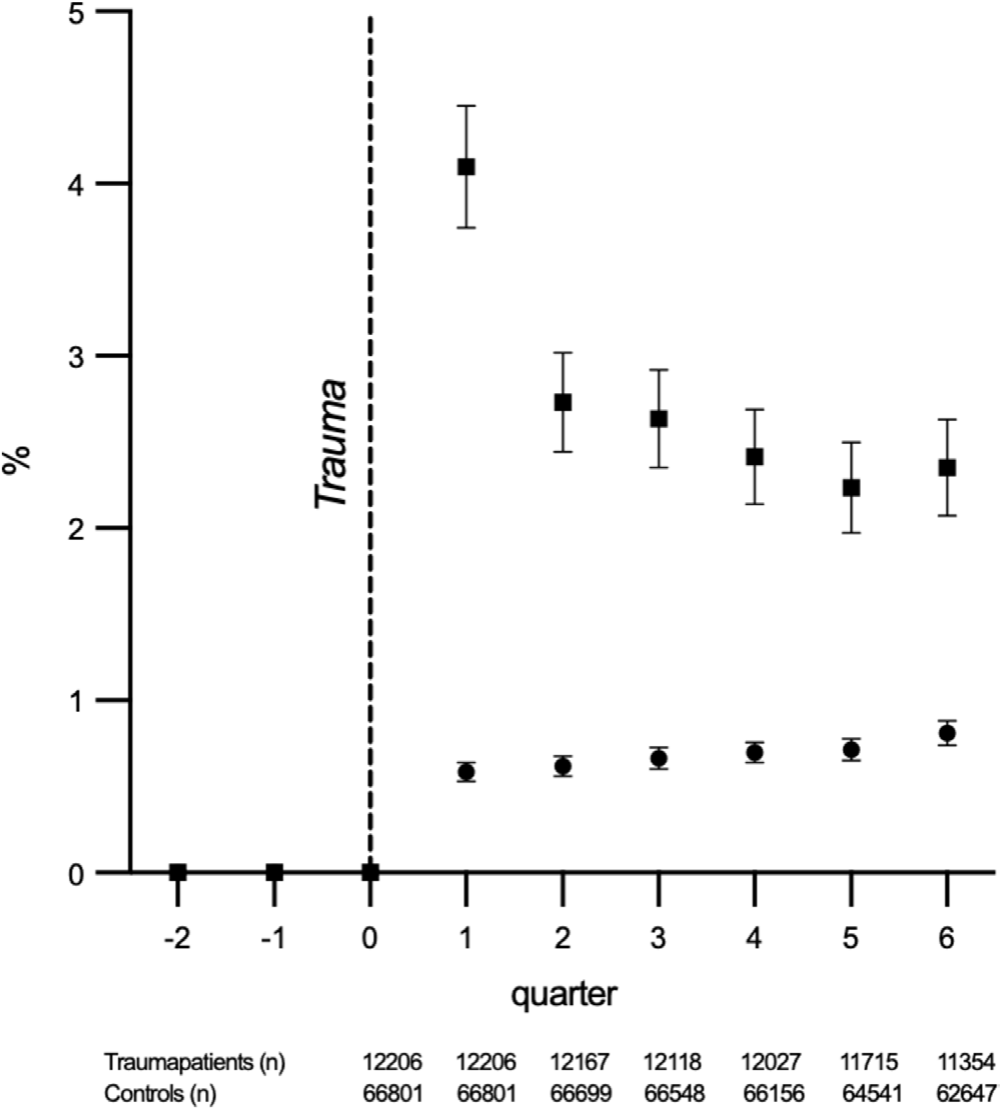
Proportion of BZD‐naïve trauma patients surviving ≥ 3 months post‐trauma and matched controls filling at least one high‐potency BZD prescription per quarter, spanning two quarters before (Q‐2, Q‐1) and six quarters after the index trauma (Q1–Q6). The proportion at each time point represents individuals filling ≥ 1 prescription in that specific quarter. The dashed vertical line indicates the time of trauma. Squares indicate trauma patients; circles indicate controls. Numbers at risk are shown below the figure.

The study included 12,206 BZD‐naive trauma patients and 66,801 matched controls (see Figure 1). Baseline characteristics are summarized in Table 1. Compared with controls, trauma patients had a higher prevalence of psychiatric comorbidity (15.0% vs. 6.7%), substance abuse (14.1% vs. 2.9%), pre‐traumatic opioid use (8.0% vs. 4.7%), and pre‐traumatic sedative‐hypnotic drug use (6.0% vs. 4.0%). Figure 2 shows the proportion of participants filling at least one BZD prescription per quarter over the 18‐month follow‐up period; numbers at risk per quarter are provided below the figure, showing that a considerable proportion of trauma patients continued BZD use throughout the follow‐up period.

**TABLE 1:** General characteristics for trauma patients and controls in the study cohort.

	Trauma patients	Matched controls
Count	12,206	66,801
Age categories, count (%)		
15–44	7297 (59.8)	38,213 (57.2)
45–54	1900 (15.6)	10,172 (15.2)
55–64	1392 (11.4)	7911 (11.8)
65–74	884 (7.2)	5270 (7.9)
75–84	464 (3.8)	3091 (4.6)
≥ 85	269 (2.2)	2144 (3.2)
Male, count (%)	8452 (69.2)	46,002 (68.9)
Income level		
Low	6004 (50.9)	29,808 (46.2)
Medium	4961 (42.0)	28,039 (43.4)
High	834 (7.1)	6718 (10.4)
Education level, count (%)		
Low	3534 (30.3)	13,784 (21.8)
Medium	5281 (45.3)	26,913 (42.6)
High	2834 (24.3)	22,546 (35.6)
CCI categories, count (%)		
CCI 0	10,067 (82.5)	57,701 (86.4)
CCI 1	1180 (9.7)	4653 (7.0)
CCI > 1	959 (7.9)	4447 (6.7)
Psychiatric comorbidity, count (%)	1825 (15.0)	4493 (6.7)
Substance abuse, count (%)	1718 (14.1)	1917 (2.9)
Pre‐traumatic opioid use, count (%)	974 (8.0)	3123 (4.7)
Pre‐traumatic low potency bdz use, count (%)	731 (6.0)	2671 (4.0)
ISS categories, count (%)		
0–8	7324 (60.0)	
9–15	2681 (22.0)	
16–24	1257 (10.3)	
25–40	766 (6.3)	
> 40	178 (1.5)	
Severe head injury^a^, count (%)	1664 (13.6)	
Severe thoracic injury^a^, count (%)	1569 (12.9)	
Severe abdominal injury^a^, count (%)	410 (3.4)	
Severe spinal injury^a^, count (%)	527 (4.3)	
Severe injury lower extremity^a^, count (%)	927 (7.6)	
Severe injury upper extremity^a^, count (%)	134 (1.1)	
Penetrating trauma, count (%)	934 (7.7)	
Shock on arrival^b^, count (%)	200 (1.6)	
Length of stay hospital, days		
0–2	7658 (62.7)	
3–7	2118 (17.4)	
> 7	2430 (19.9)	
GCS, count (%)		
13–15	10,876 (89.8)	
9–12	484 (4.0)	
3–8	750 (6.2)	
ICU admission, count (%)	2256 (18.5)	

*Note:* Categorical parameters are presented as *n* (%).

Abbreviations: bdz, benzodiazepine; CCI, Charlson comorbidity index; GCS, Glasgow coma scale; ICU, intensive care unit; ISS, injury severity score.

^a^ Severe injury equal to abbreviated injury scale (AIS) score > 2.

^b^ Shock on arrival equal to SAP (systolic arterial pressure) < 90 mmHg on arrival to the trauma unit.

In the univariate logistic regression analysis, exposure to trauma was strongly associated with initiation of high‐potency BZD use (unadjusted Odds Ratio (OR) 6.5, 95% confidence interval (CI) 5.8–7.3, *p* < 0.001, Table 2). This association remained robust in the model adjusted for age and sex (OR 6.5, 95% CI 5.8–7.3, *p* < 0.001) and persisted after full adjustment for socioeconomic status, comorbidities, and substance use (fully adjusted OR 5.4, 95% CI 4.7–6.2, *p* < 0.001, Table 2).

**TABLE 2:** Associations between exposure to trauma and post‐traumatic benzodiazepines use among trauma patients and controls, presented as OR (95% CI).

	OR (95% CI)	*p*
Unadjusted	6.5 (5.8–7.3)	< 0.001
Restricted model^a^	6.5 (5.8–7.3)	< 0.001
Full model^b^	5.4 (4.7–6.2)	< 0.001

Abbreviations: CI, confidence interval; OR, odds ratio.

^a^ Restricted model: adjusted for age and sex (variables used in matching).

^b^ Full model: in addition to the restricted model adjusted for level of income and education, somatic comorbidity, psychiatric comorbidity, substance abuse, pre‐traumatic opioid use and pre‐traumatic sedative‐hypnotic drug use.

Among trauma patients, 681 (5.6%) initiated new high‐potency BZD use within 6 months post‐injury. In the control population, 687 (1.0%) initiated such use. Comparisons between BZD users and non‐users are presented in Table S2. New users were typically older, had lower income levels, and exhibited a higher prevalence of somatic (CCI > 1: 13.7% vs. 7.5%), psychiatric (30.4% vs. 14.0%), and substance‐use comorbidity (27.0% vs. 13.3%) compared with non‐users. They also sustained more severe injuries (ISS > 8: 61.8% vs. 38.7%).

Pre‐existing substance use was more common among new BZD users, with 25% reporting substance abuse and 19% having filled prescriptions for sedative‐hypnotic drugs before the trauma. Severe injuries involving multiple regions, particularly the head, thorax, abdomen, spine, and lower extremities, were more frequent in subsequent BZD users.

Multivariable logistic regression analysis identified several independent predictors of new high‐potency BZD use (Table 3). Increasing age was associated with higher odds, as were psychiatric comorbidity (OR 2.09, 95% CI 1.70–2.58), substance abuse (OR 1.56, 95% CI 1.26–1.94), pre‐traumatic opioid use (OR 1.85, 95% CI 1.46–2.36), and pre‐traumatic sedative‐hypnotic drug use (OR 2.21, 95% CI 1.72–2.83). Trauma‐related factors such as penetrating trauma, higher injury severity scores, and prolonged hospital stays were also independently associated with increased odds of new high‐potency BZD use.

**TABLE 3:** Univariate and multivariable logistic regression analyses, associations with benzodiazepine use for trauma patients presented as OR (95% CI).

	Univariate	*p*	Multivariable	*p*
Age categories				
15–44	Ref.		Ref.	
45–54	1.39 (1.12–1.74)	0.003	1.11 (0.87–1.41)	0.40
55–64	1.72 (1.37–2.17)	< 0.001	1.36 (1.05–1.75)	0.019
65–74	2.04 (1.57–2.65)	< 0.001	1.48 (1.09–2.00)	0.013
75–84	2.31 (1.66–3.21)	< 0.001	1.76 (1.18–2.62)	0.006
≥ 85	2.87 (1.94–4.24)	< 0.001	2.32 (1.41–3.83)	0.001
Male gender	0.93 (0.79–1.10)	0.41	0.92 (0.76–1.11)	0.40
Education level				
Low	Ref.		Ref.	
Medium	0.87 (0.72–1.04)	0.14	0.96 (0.79–1.17)	0.67
High	0.96 (0.78–1.19)	0.73	1.15 (0.91–1.45)	0.26
Income level				
Low	Ref.		Ref.	
Medium	0.69 (0.59–0.81)	< 0.001	1.02 (0.84–1.25)	0.81
High	0.50 (0.34–0.73)	< 0.001	0.76 (0.50–1.17)	0.21
CCI categories				
CCI 0	Ref.		Ref.	
CCI 1	1.96 (1.58–2.44)	< 0.001	1.20 (0.94–1.54)	0.15
CCI > 1	2.14 (1.69–2.69)	< 0.001	1.21 (0.92–1.60)	0.18
Psychiatric comorbidity	2.67 (2.25–3.17)	< 0.001	2.09 (1.70–2.58)	< 0.001
Substance abuse	2.41 (2.02–2.88)	< 0.001	1.56 (1.26–1.94)	< 0.001
Pre‐trauma opioid use	2.41 (1.94–2.98)	< 0.001	1.85 (1.46–2.36)	< 0.001
Pre‐trauma sedative‐hypnotic drug use	4.00 (3.24–4.95)	< 0.001	2.21 (1.72–2.83)	< 0.001
Penetrating trauma	1.67 (1.31–2.13)	< 0.001	1.88 (1.43–2.46)	< 0.001
Shock on arrival^a^	2.83 (1.88–4.25)	< 0.001	1.20 (0.75–1.90)	0.45
ISS categories, count (%)				
0–8	Ref.		Ref.	
9–15	1.87 (1.54–2.28)	< 0.001	1.25 (0.97–1.60)	0.080
16–24	2.81 (2.24–3.53)	< 0.001	1.50 (1.10–2.05)	0.010
25–40	4.17 (3.28–5.32)	< 0.001	1.89 (1.33–2.69)	< 0.001
> 40	5.07 (3.33–7.73)	< 0.001	2.26 (1.32–3.85)	0.003
GCS				
13–15	Ref.		Ref.	
9–12	2.10 (1.54–2.85)	< 0.001	1.06 (0.75–1.50)	0.74
3–8	2.07 (1.61–2.67)	< 0.001	0.82 (0.60–1.12)	0.21
Length of stay hospital, days				
0–2	Ref.		Ref.	
3–7	1.90 (1.52–2.36)	< 0.001	1.40 (1.07–1.83)	0.014
> 7	4.27 (3.59–5.08)	< 0.001	2.80 (2.13–3.69)	< 0.001
ICU admission	2.60 (2.20–3.07)	< 0.001	1.09 (0.85–1.40)	0.48

*Note:* Categorical parameters are presented as *n* (%).

Abbreviations: bdz, benzodiazepine; CCI, Charlson comorbidity index; GCS, Glasgow coma scale; ICU, intensive care unit; ISS, injury severity score.

^a^ Shock on arrival equal to SAP (systolic arterial pressure) < 90 mmHg on arrival to the trauma unit.

A sensitivity analysis accounting for non‐random dropout due to death demonstrated that a hospital stay of three to 7 days was no longer significantly associated with new BZD use, whereas the associations for prolonged hospitalization (> 7 days) and for psychosocial factors remained stable.

During the 6‐ to 18‐month follow‐up period, new high‐potency BZD use was associated with markedly elevated mortality among trauma patients, a finding that reflects the complex clinical and psychosocial vulnerability of this group rather than implying a causal effect of BZD use. In the unadjusted Cox regression analysis, the hazard ratio (HR) for mortality was 5.3 (95% CI 3.7–7.6, *p* < 0.001). This association attenuated but remained statistically significant after adjustment for age, sex, somatic and psychiatric comorbidity, substance abuse, and ISS (adjusted HR 2.9, 95% CI 2.0–4.2, *p* < 0.001). A sensitivity analysis additionally adjusting for severe AIS‐region injuries yielded a consistent estimate (adjusted HR 3.1, 95% CI 2.1–4.4, *p* < 0.001). Results were robust when deaths within 6 months (rather than 3 months) were used as the exclusion criterion (fully adjusted OR 5.5, 95% CI 4.8–6.3, *p* < 0.001).

## 4 Discussion

This large, population‐based cohort study indicates that trauma exposure is independently associated with new use of high‐potency benzodiazepines (BZDs) among individuals without prior BZD prescriptions. After adjustment for demographic, socioeconomic, and clinical factors, trauma patients had more than a fivefold increased likelihood of initiating such treatment compared with matched, uninjured controls. Furthermore, new BZD use was associated with markedly elevated long‐term mortality, an association that most plausibly reflects the underlying vulnerability profile of BZD initiators rather than a direct causal effect as discussed below.

The observed association between trauma exposure and subsequent BZD initiation, despite adjustment for extensive covariates, suggests that trauma itself may serve as a precipitating factor for pharmacological management of psychological or physical distress. Although short‐term BZD prescriptions may sometimes be clinically justified, such as for acute anxiety, insomnia, or agitation, our findings highlight a potential risk for use extending beyond the intended acute phase [23, 24]. The ongoing use of high‐potency BZD 18 months after trauma suggests that initial prescriptions can evolve into chronic treatment patterns, raising concerns about dependency and adverse health effects.

Within the trauma cohort, several independent predictors of new high‐potency BZD use were identified. These included increasing age, psychiatric comorbidity, substance abuse, pre‐traumatic opioid or sedative‐hypnotic drug use, and markers of greater injury severity. This pattern supports the notion that psychosocial vulnerability and pre‐existing pharmacological coping behaviors contribute substantially to the initiation of high‐potency BZD use following trauma.

The strong associations with psychiatric and substance‐use comorbidities are consistent with prior studies linking psychological distress and maladaptive coping to increased sedative–hypnotic use [25, 26]. Individuals with a history of sedative‐hypnotic drug or opioid use may represent a subgroup accustomed to pharmacological management of stress or pain, making them more susceptible to transitioning to increased use after trauma [27]. The link between pre‐traumatic opioid use and post‐trauma BZD initiation also underscores the clinical overlap between pain management and mental health challenges, a combination associated with increased risk of adverse outcomes, including overdose and dependence [28, 29].

Injury‐related factors such as penetrating trauma, higher injury severity scores, and prolonged hospital stays were also associated with increased odds of new BZD use. These findings may reflect both the clinical complexity of such cases and extended exposure to pharmacological interventions during recovery [30]. Older age further amplified risk, potentially reflecting a higher baseline prevalence of sleep disturbances, anxiety, or polypharmacy in this group, which may facilitate continued prescribing [31]. It should also be emphasized that the use of benzodiazepines in older patients is generally discouraged due to the higher risk of adverse effects compared with younger individuals [32].

New high‐potency BZD use was associated with a nearly threefold increase in long‐term mortality; however, this association cannot be interpreted as causal. The elevated mortality most plausibly reflects the complex clinical and psychosocial vulnerability of patients who initiate BZD prescriptions. Several mechanisms may nonetheless contribute. Benzodiazepines have been linked to cognitive impairment, falls, and accidents, as well as increased risk of respiratory depression, particularly when co‐prescribed with opioids [28, 29, 33]. Additionally, prior research has shown associations between long‐term BZD use and elevated risks of overdose and suicide [34, 35]. The initiation of BZD therapy following trauma may thus serve as a marker for patients with complex psychosocial or clinical profiles who face a higher baseline risk of adverse outcomes.

The findings underscore the need for cautious prescribing of BZDs following trauma, especially in patients with pre‐existing psychiatric or substance use disorders. Given that chronic BZD use does not confer long‐term benefit and carries well‐documented risks, clinicians should prioritize non‐pharmacological interventions and, when pharmacological treatment is deemed necessary, adhere to strictly limited prescription practices. Screening for psychosocial risk factors at the time of trauma admission may help identify individuals at elevated risk for developing misuse. Early involvement of multidisciplinary care teams, including mental health professionals, may reduce reliance on sedatives and promote safer, more individualized treatment strategies.

### 4.1 Strengths and Limitations

Major strengths of this study include its large, population‐based design, use of validated national registers, and the ability to link clinical and socioeconomic data at the individual level. The focus on BZD‐naive patients strengthens the inference of new onset following trauma. However, some limitations should be acknowledged. First, prescription data reflect medication dispensing rather than confirmed intake, which may introduce misclassification bias. Second, residual confounding cannot be excluded. Although we adjusted for ISS and, in a sensitivity analysis, for severe AIS‐region injuries, important severity markers, including physiological parameters (e.g., base excess, lactate) and probability‐of‐survival scores (e.g., TRISS), were not available. The substantial attenuation of the mortality HR from 5.3 (unadjusted) to 2.9 (ISS‐adjusted) underscores the contribution of measured confounders, and further unmeasured confounders are likely to exist. Third, the cohort originates from a regional trauma center in Sweden; while healthcare access is universal, prescribing patterns may differ across settings, potentially affecting generalizability.

### 4.2 Conclusion

In summary, trauma exposure was independently associated with new high‐potency BZD use among previously BZD‐naive individuals. Pre‐existing psychiatric comorbidity, substance use, and greater injury severity emerged as key predictors. New BZD use was further associated with increased long‐term mortality. These findings highlight the importance of careful prescribing, close follow‐up, and development of alternative treatment approaches for post‐traumatic distress to reduce the risk of chronic sedative use and its associated adverse outcomes.

## Author Contributions

**Anders Oldner:** conceptualization, methodology, supervision, writing – review and editing. **Mikael Eriksson:** conceptualization, methodology, writing – review and editing. **Emma Larsson:** data curation, formal analysis, writing – original draft. **Jesper Eriksson:** conceptualization, methodology, project administration, writing – review and editing. **Erik von Oelreich:** conceptualization, methodology, project administration, writing – review and editing. Jesper Eriksson and Erik von Oelreich contributed equally to this work. All authors read and approved the final manuscript.

## Funding

This work was supported by Stockholms Läns Landsting, FoUI‐985563 Stockholm läns landsting, Stiftelsen Tornspiran, 820 Carnegie, Svenska Läkaresällskapet.

## Conflicts of Interest

The authors declare no conflicts of interest.

## Supporting information


**Data S1:** Benzodiazepine initiation after trauma and associated patient characteristics.
**Table S1:** Anatomical Therapeutic Chemical (ATC) classification codes and definition of benzodiazepines and related GABA‐A agonists or sedative‐hypnotic drugs.
**Table S2:** Baseline characteristics of trauma patients stratified by post‐traumatic benzodiazepine initiation.

## Data Availability

The data that support the findings of this study are available on request from the corresponding author. The data are not publicly available due to privacy or ethical restrictions.

## References

[1] Diseases GBD and Injuries C , “Global Burden of 369 Diseases and Injuries in 204 Countries and Territories, 1990‐2019: A Systematic Analysis for the Global Burden of Disease Study 2019,” Lancet 396 (2020): 1204–1222, 10.1016/S0140-6736(20)30925-9. 33069326 PMC7567026

[2] J. C. Brooks , D. J. Strauss , R. M. Shavelle , et al., “Long‐Term Disability and Survival in Traumatic Brain Injury: Results From the National Institute on Disability and Rehabilitation Research Model Systems,” Archives of Physical Medicine and Rehabilitation 94 (2013): 2203–2209, 10.1016/j.apmr.2013.07.005. 23872079

[3] E. B. Powelson , B. Mills , W. Henderson‐Drager , M. Boyd , M. S. Vavilala , and M. Curatolo , “Predicting Chronic Pain After Major Traumatic Injury,” Scandinavian Journal of Pain 19 (2019): 453–464, 10.1515/sjpain-2019-0040. 31116704

[4] J. P. Herrera‐Escobar , S. S. Al Rafai , A. J. Seshadri , et al., “A Multicenter Study of Post‐Traumatic Stress Disorder After Injury: Mechanism Matters More Than Injury Severity,” Surgery 164 (2018): 1246–1250, 10.1016/j.surg.2018.07.017. 30170820

[5] T. M. Bell , A. N. Vetor , and B. L. Zarzaur , “Prevalence and Treatment of Depression and Posttraumatic Stress Disorder Among Trauma Patients With Non‐Neurological Injuries,” Journal of Trauma and Acute Care Surgery 85 (2018): 999–1006, 10.1097/TA.0000000000001992. 29851909 PMC6202214

[6] A. N. Edinoff , C. A. Nix , J. Hollier , et al., “Benzodiazepines: Uses, Dangers, and Clinical Considerations,” Neurology International 13 (2021): 594–607, 10.3390/neurolint13040059. 34842811 PMC8629021

[7] K. M. Kennedy and J. O'Riordan , “Prescribing Benzodiazepines in General Practice,” British Journal of General Practice 69 (2019): 152–153, 10.3399/bjgp19X701753. PMC6400612 30819759

[8] M. Soyka , I. Wild , B. Caulet , et al., “Long‐Term Use of Benzodiazepines in Chronic Insomnia: A European Perspective,” Frontiers in Psychiatry 14 (2023): 1212028, 10.3389/fpsyt.2023.1212028. 37599882 PMC10433200

[9] J. Brett and B. Murnion , “Management of Benzodiazepine Misuse and Dependence,” Australian Prescriber 38 (2015): 152–155, 10.18773/austprescr.2015.055. 26648651 PMC4657308

[10] C. Kruger , M. H. Lindberg , S. Burmester , et al., “Experiences of Long‐Term Benzodiazepine Use and Addiction Amid Changes in Guidelines for the Prescription of Narcotic Drugs: A Qualitative Study,” BMC Public Health 25 (2025): 2652, 10.1186/s12889-025-23920-9. 40764568 PMC12326687

[11] M. Lader , “Benzodiazepines Revisited – Will We Ever Learn?,” Addiction 106 (2011): 2086–2109, 10.1111/j.1360-0443.2011.03563.x. 21714826

[12] C. Corral‐Tuesta , A. Rodriguez Diaz‐Pavon , B. Montero‐Errasquin , et al., “Chronic Benzodiazepine Usage Among Older People: Prevalence, Indications, and Treatment Modifications in Patients Admitted to an Acute Geriatric Unit,” European Geriatric Medicine 15 (2024): 539–543, 10.1007/s41999-023-00918-3. 38214865

[13] K. Mansoor , B. De Souza Goncalves , H. V. Lakhani , et al., “Prevalence of Substance Abuse Among Trauma Patients in Rural West Virginia,” Cureus 15 (2023): e36468, 10.7759/cureus.36468. 37090413 PMC10117230

[14] J. H. Olson‐Madden , L. A. Brenner , J. D. Corrigan , et al., “Substance Use and Mild Traumatic Brain Injury Risk Reduction and Prevention: A Novel Model for Treatment,” Rehabilitation Research and Practice 2012 (2012): 174579, 10.1155/2012/174579. 22685663 PMC3363008

[15] M. D. Horner , P. L. Ferguson , A. W. Selassie , L. Labbate , K. Kniele , and J. Corrigan , “Patterns of Alcohol Use 1 Year After Traumatic Brain Injury: A Population‐Based, Epidemiological Study,” Journal of the International Neuropsychological Society: JINS 11 (2005): 322–330, 10.1017/S135561770505037X. 15892908

[16] S. Weich , H. L. Pearce , P. Croft , et al., “Effect of Anxiolytic and Hypnotic Drug Prescriptions on Mortality Hazards: Retrospective Cohort Study,” BMJ (Clinical Research Ed.) 348 (2014): g1996, 10.1136/bmj.g1996. PMC3959619 24647164

[17] E. von Elm , D. G. Altman , M. Egger , S. J. Pocock , P. C. Gøtzsche , and J. P. Vandenbroucke , “The Strengthening the Reporting of Observational Studies in Epidemiology (STROBE) Statement: Guidelines for Reporting Observational Studies,” International Journal of Surgery 12 (2014): 1495–1499, 10.1016/j.ijsu.2014.07.013. 25046131

[18] J. F. Ludvigsson , P. Otterblad‐Olausson , B. U. Pettersson , et al., “The Swedish Personal Identity Number: Possibilities and Pitfalls in Healthcare and Medical Research,” European Journal of Epidemiology 24 (2009): 659–667, 10.1007/s10654-009-9350-y. 19504049 PMC2773709

[19] B. Wettermark , N. Hammar , C. M. Fored , et al., “The New Swedish Prescribed Drug Register – Opportunities for Pharmacoepidemiological Research and Experience From the First Six Months,” Pharmacoepidemiology and Drug Safety 16 (2007): 726–735, 10.1002/pds.1294. 16897791

[20] J. F. Ludvigsson , P. Svedberg , O. Olen , G. Bruze , and M. Neovius , “The Longitudinal Integrated Database for Health Insurance and Labour Market Studies (LISA) and Its Use in Medical Research,” European Journal of Epidemiology 34 (2019): 423–437, 10.1007/s10654-019-00511-8. 30929112 PMC6451717

[21] H. L. Brooke , M. Talbäck , J. Hörnblad , et al., “The Swedish Cause of Death Register,” European Journal of Epidemiology 32 (2017): 765–773, 10.1007/s10654-017-0316-1. 28983736 PMC5662659

[22] D. Scharfstein , A. Rotnitzky , and J. Robins , “Adjusting for Nonignorable Drop‐Out Using Semiparametric Nonresponse Models,” Journal of the American Statistical Association 94 (1999): 1096–1120.

[23] M. Lader , “Benzodiazepine Harm: How Can It Be Reduced?,” British Journal of Clinical Pharmacology 77 (2014): 295–301, 10.1111/j.1365-2125.2012.04418.x. 22882333 PMC4014015

[24] B. Dell'osso and M. Lader , “Do Benzodiazepines Still Deserve a Major Role in the Treatment of Psychiatric Disorders? A Critical Reappraisal,” European Psychiatry 28 (2013): 7–20, 10.1016/j.eurpsy.2011.11.003. 22521806

[25] A. Schmitz , “Benzodiazepine Use, Misuse, and Abuse: A Review,” Mental Health Clinician 6 (2016): 120–126, 10.9740/mhc.2016.05.120. 29955458 PMC6007645

[26] T. W. Rosenqvist , M. K. Wium‐Andersen , I. K. Wium‐Andersen , et al., “Long‐Term Use of Benzodiazepines and Benzodiazepine‐Related Drugs: A Register‐Based Danish Cohort Study on Determinants and Risk of Dose Escalation,” American Journal of Psychiatry 181 (2024): 246–254, 10.1176/appi.ajp.20230075. 37727098

[27] G. Chouinard , “Issues in the Clinical Use of Benzodiazepines: Potency, Withdrawal, and Rebound,” Journal of Clinical Psychiatry 65, no. 5 (2004): 7–12. 15078112

[28] J. D. Jones , S. Mogali , and S. D. Comer , “Polydrug Abuse: A Review of Opioid and Benzodiazepine Combination Use,” Drug and Alcohol Dependence 125 (2012): 8–18, 10.1016/j.drugalcdep.2012.07.004. 22857878 PMC3454351

[29] C. Day , Benzodiazepines in Combination With Opioid Pain Relievers or Alcohol: Greater Risk of More Serious ED Visit Outcomes. The CBHSQ Report (Substance Abuse and Mental Health Services Administration (SAMHSA), 2013), 1–9. 27631051

[30] C. M. Bell , H. D. Fischer , S. S. Gill , et al., “Initiation of Benzodiazepines in the Elderly After Hospitalization,” Journal of General Internal Medicine 22 (2007): 1024–1029, 10.1007/s11606-007-0194-4. 17453266 PMC2330138

[31] L. B. Gerlach , I. R. Wiechers , and D. T. Maust , “Prescription Benzodiazepine Use Among Older Adults: A Critical Review,” Harvard Review of Psychiatry 26 (2018): 264–273, 10.1097/HRP.0000000000000190. 30188338 PMC6129989

[32] Finnish , “WgsubtFMSDatFSRSHTFMSD. Insomnia. Current Care Guidelines,” (2015).

[33] M. A. Bachhuber , S. Hennessy , C. O. Cunningham , et al., “Increasing Benzodiazepine Prescriptions and Overdose Mortality in the United States, 1996‐2013,” American Journal of Public Health 106 (2016): 686–688, 10.2105/AJPH.2016.303061. 26890165 PMC4816010

[34] T. J. Dodds , “Prescribed Benzodiazepines and Suicide Risk: A Review of the Literature,” Primary Care Companion CNS Disorders 19 (2017): 20170302, 10.4088/PCC.16r02037. 28257172

[35] V. Cato , F. Hollandare , A. Nordenskjold , et al., “Association Between Benzodiazepines and Suicide Risk: A Matched Case‐Control Study,” BMC Psychiatry 19 (2019): 317, 10.1186/s12888-019-2312-3. 31655565 PMC6815437

