# Supplementary material for: Initiation of High‐Potency Benzodiazepine Prescriptions Among Survivors of Severe Trauma
Source: Acta Anaesthesiol Scand. 2026 Apr 30;70:e70245. doi: 10.1111/aas.70245 (PMC13133234; doi:10.1111/aas.70245)
Supplement: Supplementary file 1 — Data S1: Benzodiazepine initiation after trauma and associated patient characteristics. Table S1: Anatomical Therapeutic Chemical (ATC) classification codes and definition of benzodiazepines and related GABA‐A agonists or sedative‐hypnotic drugs. Table S2: Baseline characteristics of trauma patients stratified by post‐traumatic benzodiazepine initiation. [file AAS-70-0-s001.docx]

**x**

Supplemental Digital Content

Table S1 3

Table S2 4

# Table S1

Anatomical Therapeutic Chemical (ATC) Classification System codes starting with N03, N05 or R06 and classification as benzodiazepines and related GABA-A agonists or sedative-hypnotic drugs.

| **Benzodiazepines and related GABA-A agonists** |
| --- |
| ATC starting with:  N05BA  N05BC  N05CD  N03AE01 |
|  |
| **Sedative-hypnotic drugs** |
| ATC starting with:  N05BB  N05BE  N05CC  N05CF  N05CH  N05CM  N05CX  R06A |

# Table S2

General characteristics for trauma patients stratified by post-traumatic BZD initiation

|  | **No BZD initiation** | **Benzodiazepine use** |
| --- | --- | --- |
| **Count** | 11 525 | 681 |
| **Age categories, count (%)**  **15-44**  **45-54**  **55-64**  **65-74**  **75-84**  **≥ 85** | 6980 (60.6)  1787 (15.5)  1291 (11.2)  809 (7.0)  420 (3.6)  238 (2.1) | 317 (46.5)  113 (16.6)  101 (14.8)  75 (11.0)  44 (6.5)  31 (4.6) |
| **Male, count (%)** | 7990 (69.3) | 462 (67.8) |
| **Income level**  **Low**  **Medium**  **High** | 5599 (50.3)  4725 (42.5)  805 (7.2) | 405 (60.4)  236 (35.2)  29 (4.3) |
| **Education level, count (%)**  **Low**  **Medium**  **High** | 3321 (30.2)  5002 (45.5)  2669 (24.3) | 213 (32.4)  279 (42.5)  165 (25.1) |
| **CCI categories, count (%)**  **CCI 0**  **CCI 1**  **CCI > 1** | 9585 (83.2)  1074 (9.3)  866 (7.5) | 482 (70.8)  106 (15.6)  93 (13.7) |
| **Psychiatric comorbidity, count (%)** | 1618 (14.0) | 207 (30.4) |
| **Substance abuse, count (%)** | 1534 (13.3) | 184 (27.0) |
| **Pre-traumatic opioid use, count (%)** | 863 (7.5) | 111 (16.3) |
| **Pre-traumatic sedative-hypnotic drug use, count (%)** | 607 (5.3) | 124 (18.2) |
| **ISS categories, count (%)**  **0-8**  **9-15**  **16-24**  **25-40**  **> 40** | 7064 (61.3)  2508 (21.8)  1139 (9.9)  664 (5.8)  150 (1.3) | 260 (38.2)  173 (25.4)  118 (17.3)  102 (15.0)  28 (4.1) |
| **Severe head injury*, count (%)** | 1512 (13.1) | 152 (22.3) |
| **Severe thoracic injury*, count (%)** | 1441 (12.5) | 128 (18.8) |
| **Severe abdominal injury*, count (%)** | 368 (3.2) | 42 (6.2) |
| **Severe spinal injury*, count (%)** | 456 (4.0) | 71 (10.4) |
| **Severe injury lower extremity*, count (%)** | 830 (7.2) | 97 (14.2) |
| **Severe injury upper extremity*, count (%)** | 122 (1.1) | 12 (1.8) |
| **Penetrating trauma, count (%)** | 854 (7.4) | 80 (11.8) |
| **Shock on arrival**, count (%)** | 172 (1.5) | 28 (4.1) |
| **Length of stay hospital, days**  **0-2**  **3-7**  **>7** | 7409 (64.3)  1991 (17.3)  2125 (18.4) | 249 (36.6)  127 (18.6)  305 (44.8) |
| **GCS, count (%)**  **13-15**  **9-12**  **3-8** | 10 322 (90.3)  435 (3.8)  675 (5.9) | 554 (81.7)  49 (7.2)  75 (11.1) |
| **ICU admission, count (%)** | 2014 (17.5) | 242 (35.5) |

Categorical parameters are presented as n (%). CCI, Charlson Comorbidity Index; ISS, Injury Severity Score; *Severe injury equal to Abbreviated Injury Scale (AIS) score > 2; **Shock on arrival equal to SAP (Systolic Arterial Pressure) < 90 mmHg on arrival to the trauma unit; GCS, Glasgow Coma Scale; ICU, Intensive Care Unit.
